# Mining and Characterization of Sequence Tagged Microsatellites from the Brown Planthopper *Nilaparvata lugens*

**DOI:** 10.1673/031.011.13401

**Published:** 2011-10-04

**Authors:** Jing-Tao Sun, Yan-Kai Zhang, Cheng Ge, Xiao-Yue Hong

**Affiliations:** Department of Entomology, Nanjing Agricultural University, Nanjing, Jiangsu 210095, China

**Keywords:** migration routine, expressed sequences, population genetics

## Abstract

The brown planthopper, *Nilaparvata lugens* (Stål) (Hemiptera: Delphacidae), is an important pest of rice. To better understand the migration pattern and population structure of the Chinese populations of *N. lugens*, we developed and characterized 12 polymorphic microsatellites from the expressed sequence tags database of *N. lugens.* The occurrence of these simple sequence repeats was assessed in three populations collected from three provinces of China. The number of alleles per locus ranged from 3 to 13 with an average of 6.5 alleles per locus. The mean observed heterozygosity of the three populations ranged from 0.051 to 0.772 and the expected heterozygosity ranged from 0.074 to 0.766. The sequences of the 12 markers were highly variable. The polymorphism information content of the 12 markers was high and ranged from 0.074 to 0.807 (mean = 0.503). Sequencing of microsatellite alleles revealed that the fragment length differences were mainly due to the variation of the repeat motif. Significant genetic differentiation was detected among the three *N. lugens* populations as the *F*st ranged from 0.034 to 0.273. Principle coordinates analysis also revealed significant genetic differentiation between populations of different years. We conclude that these microsatellite markers will be a powerful tools to study the migration routine of the *N. lugens*.

## Introduction

The brown planthopper, *Nilaparvata lugens* (Stål) (Hemiptera: Delphacidae) is an important pest of rice. It can cause hopperburn, that is characterized by complete wilting and drying of affected plants. It also transmits two rice viruses, grassy stunt and ragged stunt. *N. lugens* migrates northwards or north-eastwards from tropical and subtropical areas each year, in a series of distinct, windborne movements, progressively infesting the summer rice crop (Cheng et al. 1979). The origin of these migrations and the genetic diversity of the pest are not well known. Recent plant studies using informative genetic markers (i.e., microsatellites) have effectively revealed pollen dispersal mechanisms by analyzing the population genetic structure of reproductive trees and parentage of seedlings (Geng et al. 2008; Isagi et al. 2007). Therefore, combining sufficient microsatellite markers and direct observation methods, such as, airbone net-traps or radar, may offer additional insights into the *N. lugens* migration mechanism.

Microsatellites or simple sequence repeats (SSR) are tandemly repeated motifs of 1–6 bases found in nearly all prokaryotic and eukaryotic genomes. They are present in both coding and non-coding regions and are often characterized by a high degree of length polymorphism (Zane et al. 2002). The cause of such polymorphisms is still under debate though it appears most likely to be slippage events during DNA replication (Schlotterer et al. 1992). SSR markers have a number of characteristics that make them well suited for population genetic studies, genome mapping and marker-assisted breeding. These characteristics include a high level of polymorphism, codominant Mendelian inheritance, a high frequency of occurrence, and ease of detection by PCR (Valdes et al. 1993; Akkaya et al. 1995; Schuler et al. 1996). Currently, only a small number of microsatellite markers have been described in the *N. lugens* (Molecular Ecology Resources Primer Development Consortium et al. 2009; Mun et al. 2004). A variety of methods for SSR isolation have been developed in recent years. The efforts required to obtain sufficient amounts of SSR primer pairs have been comprehensively reviewed by Zane et al. (2002) and Squirrell et al. (2003). However, the conventional strategies used to develop SSR markers are usually labor-intensive, time-consuming and expensive.

An enormous number of ESTs are now available in the public sequence database, and can be exploited to identify markers inexpensively. Compared with conventional markers derived from genomic DNA, EST-derived markers are easier to develop, more informative, and highly transferable. In this study, existing *N. lugens* ESTs were mined for new microsatellites to contribute to the study of *N. lugens* genetic diversity and migration routes.

## Materials and Methods

### Data mining

37, 348 ESTs of the *N. lugens* available as of June 2008 were downloaded from GenBank (www.ncbi.nlm.nih.gov/dbEST). The EST-trimmer software (www.pgrc.ipkgatersleben.de/misa/download/est_trimmer.pl) was first used to remove the 5′or 3′ end of polyA or polyT stretches until there were no (A)_5_ or (T)_5_ within the range of 50 bp, EST sequences less than 100 bp in length were discarded while only 700 bp on the 5′ end were retained for ESTs greater than 700bp in length. CAP3 software was used to assemble those sequences using the default values (Huang et al. 1999).

The obtained unigenes containing perfect SSRs were identified by the MISA software (www.pgrc.ipk-gatersleben.de/misa) and the following parameters were adopted: at least six repeats for di-, five repeats for tri-, tetra-, penta-, and hexanucleotidic. To obtain an idea about the putative functions of SSR containing genes, these sequences were compared to the nonredundant (nr) protein database of the NCBI Database (www.ncbi.nlm.nih.gov/blast) using 1e-07 as the cutoff expected value. 180 unigenes containing SSR from the unknown gene group derived from the blast were selected randomly for primer design using Primer 5.0 (www.premierbiosoft.com). The 180 unigenes selected for primer design contained only one perfect microsatellite.

### *N. lugens* sampling and DNA extraction

Three *N. lugens* populations, totaling 140 *N. lugens* adults were sampled from three provinces of China during the summer of 2008 and 2010: Guangxi Province (GX), Jiangsu Province (JS), Zhejiang Province (XJ). Information of these *N. lugens* populations is summarized in Table 1. These populations were sampled by randomly collecting adults from 20 rice plants in a 5-^×^5-m square. Genomic DNA was extracted from individual adult males while head and thorax were collected from females following the procedure of Gomi et al. (1997). Briefly, *N. lugens* individuals or head and thorax were placed in 1.5 ml microcentrifuge tubes with 25 µl of a mixture of ice-cold STE buffer (100 mM NaCl, 10 mM Tris-HCl, and 1 mM EDTA, pH 8.0) and were homogenized with a plastic pestle on ice. Proteins were removed with 2 µl of 10 mg/ml proteinase K. The mixtures were incubated at 37° C for 30 min, and proteinase K was inactivated at 95° C for 5 min. The DNA solution was then diluted by adding 75 µl sterilized deionized water to each tube. The samples were briefly centrifuged and stored at -20° C for later use.

### Primer screening and polymorphism detection

To examine the effectiveness of primer pairs designed to amplify SSR markers, 140 *N. lugens* adult sampled from three provinces of China were used separately for the template DNA extraction as described by Gomi et al. (1997). The forward primer of each set was tailed with AP2 [5′-CTATAGGGCACGCGTGGT-3′] to facilitate labeling. 20 µl of the reaction mixture contained three primers (forward primer: 0.04 µM; reverse primer: 0.2 µM; AP2 primer: 0.2 µM), 10–100 ng template DNA, 0.2 mmol-^1^ of each dNTP, 1×PCR buffer (Fermentas, Canada, www.fermentas.com), 0.25 U of DreamTaq DNA Polymerase (Fermentas). PCR amplification was conducted on Applied Biosystems Veriti Thermal Cycler (Applied Biosystems, www.appliedbiosystems.com). The cycling conditions consisted of a touchdown regime as follows: an initial denaturing step of 3 min at 95° C, followed by 20 cycles of 30 s at 95° C, 30 s at 58° C (annealing temperature was reduced by 0.5° C per cycle), 30 s at 72° C, followed by 15 cycles of 30 s at 95° C, 30 s at 48° C, 30 s at 72° C. PCR products were analyzed using ABI 3130 sequencer (Applied Biosystems) according to the manufacturer's instructions. Allele sizes were determined using GENEMAPPER version 4.0 (Applied Biosystems), using LIZ-500(-250) as size standard.

### Microsatellite sequencing

The DNA sequences of different microsatellite alleles determined by the capillary sequencer were amplified from genomic DNA by PCR in 50µl reactions with non-fluorescent labeling primers (conditions as above). PCR products were purified with a PCR Cleanup Kit (Axygen, www.axygen.com) and cloned into a TA cloning vector (Invitrogen, www.invitrogen.com). The positive clones were screened and directly sequenced. Six clones were sequenced for each individual to eliminate the errors resulting from Taq polymerase misincorporation or in vitro recombinant PCR products (Ennis et al. 1990). The ClustalX (Thompson et al. 1997) program was used to compare the amplified SSR alleles.

### Statistical analysis

Null allele frequencies were determined with Micro-Checker version 2.2.3 using the Oosterhout algorithm (van Oosterhout et al. 2004). Expected heterozygosity, observed heterozygosity and the polymorphism information content were calculated for each locus with Cervus version 3.0 (Marshall et al. 1998). Hardy-Weinberg equilibrium and genotypic linkage disequilibrium between pairs of microsatellites were calculated with Genepop 3.4 (Raymond et al. 1995); (Markov chain Monte Carlo parameters: 10,000 dememorization steps, 100 batches and 5000 iterations per batch). Sequential Bonferroni correction (Rice 1989) was applied for all multiple tests. When the hypothesis of random allele association was rejected, tests were performed using Genepop to find out whether deviations were the result of a deficit or an excess of heterozygotes. In order to detect the genetic differentiation of the three *N. lugens* populations, pairwise *F*ST values for each population comparison were calculated with FSTAT2.9.3.2 software (Goudet 2002). We also performed a principal coordinates analysis of the 140 individuals using GenAlex 6 software (Peakall Souse. 2006). Input for principle coordinates analysis consisted of individual pairwise genetic distance matrices of the proportion of shared alleles calculated by MSAnalyzer v4.05 software (Dieringer and Schlotterer 2003).

## Results

### Data mining results

**Figure 1.**
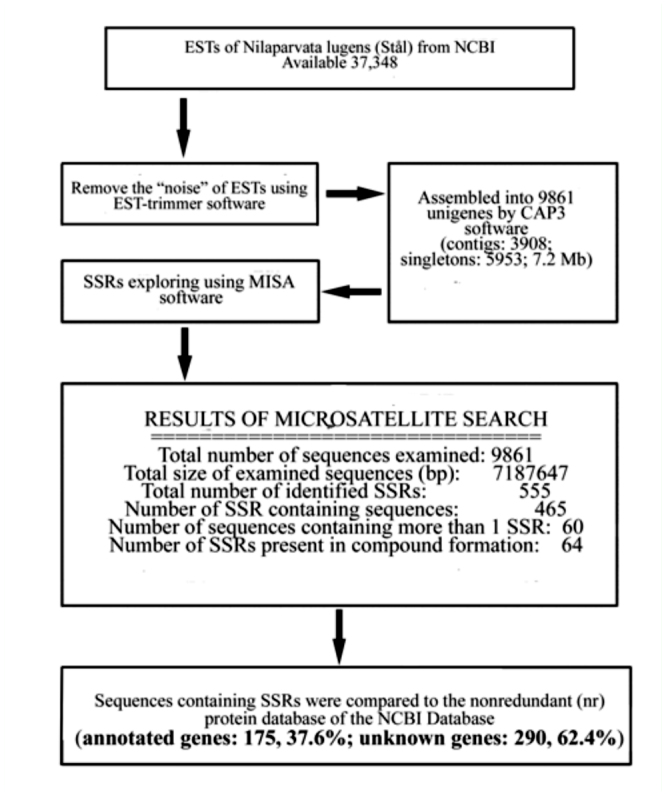
Scheme used for data exploring and development of EST-SSRs markers from *Nilaparvata lugens* ESTs. High quality figures are available online.

A total of 37,348 *N. lugens* ESTs were downloaded from GenBank, trimmed, and assembled into 9861 unigenes (3908 contigs and 5953 singletons) for a total length of 7.2 Mb. Of these unigenes, 465 were identified as containing SSR motifs by MISA. Of these 465 sequences, 60 had more than one SSR motif and 64 had compound SSRs. The 465 sequences included 175 (37.6%) sequences representing known genes and 290 (62.4%) that did not match any genes in GenBank. The above numbers are summarized in Figure 1.

### Distribution and frequencies of SSRs in *Nilaparvata lugens*

The distribution and frequencies of non-redundant EST-SSRs in the *N. lugens* transcriptome were 1 SSR/13.0 kb and most of them were smaller repeat-unit size (Table 2). Of the 555 SSR loci, 165 (29.7%) were dinucleotide repeats (DNRs). AG/CT motif was the most common among DNRs, accounting for 40.6% of the unigenes containing SSRs, followed by AT/TA (29.7%) and AC/TG (29.7%); CG/GC was not seen. The main repeat motif was trinucleotide repeats (TNRs), accounting for 65.2% of the unigenes containing SSRs. The main TNRs were AAG/TTC (30.0%) and AAT/TTA (19.1%). The frequency of other repeats motif was very low in our study, accounting for 5.0%.

### Microstatellite development and marker polymorphism

**Figure 2.**
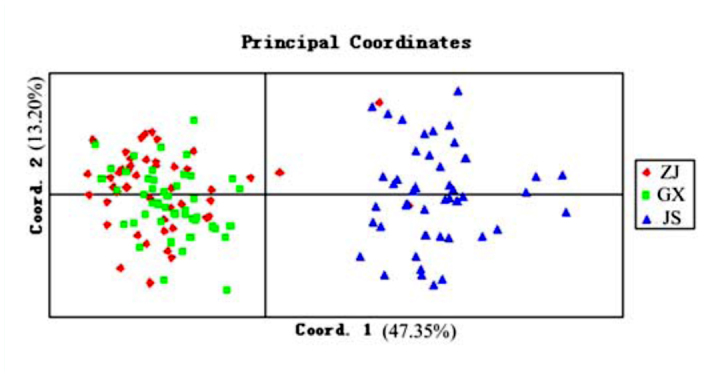
Principal coordinate analysis of genetic distance among 140 samples from three populations of *Nilaparvata lugens* in China. Coordinates 1 and 2 explain 60.50% of variation in the data. High quality figures are available online.

Of the 180 primer pairs, 76 primer pairs amplified the expected products, 98 primer pairs had no products and six primer sets yielded larger products indicating the existence of introns. Of the 76 successful primer pairs, 12 produced clean band patterns and revealed polymorphism among the tested brown planthopper genotypes. Primer sequence and general character of the 12 new microsatellite loci isolated in the *N. lugens* was summarized in Table 2. The number of alleles detected ranged from 3 to 13 per locus, with an average of 6.5 (Table 4). The observed and expected heterozygosity across the three populations ranged from 0.051 to 0.772 and from 0.074 to 0.764 respectively. The polymorphism information content was high and ranged from 0.074 to 0.807, with a mean of 0.503. After Sequential Bonferroni correction for multiple tests, NL177 displayed a significant departure from Hardy-Weinberg equilibrium (HWE) due to significant heterozygote deficiency in ZJ and JS populations. In addition, the same consequence also occurred at the locus of NL22 locus in JS population. No significant heterozygote excess was detected. No linkage disequilibrium was found between any pairs of the microsatellites. Micro—Checker 2.2.3 software revealed the probable presence of null alleles for NL22, NL121, NL162, and NL177 in the three populations. However, the null allele frequencies of the four loci in three populations were very low. Only NL177 in JS population was higher than 0.200. Pairwise estimates of *F*ST calculated between pairs of populations indicated significant genetic differentiation between populations. The biggest differentiation existed between GX and JS (*F*st = 0.280) which was similar to the differentiation between ZJ and JS (*F*st = 0.273). The GX and ZJ populations that were sampled in the same year displayed low genetic differentiation (*F*st = 0.034), but the differentiation between them was also significant (*p* < 0.05). Further principal component analysis based on genetic distance matrices of the proportion of shared alleles confirmed the genetic distinction across ZJ, GX and JS (Figure. 2).

### Microsatellite mutation patterns

**Figure 3.**
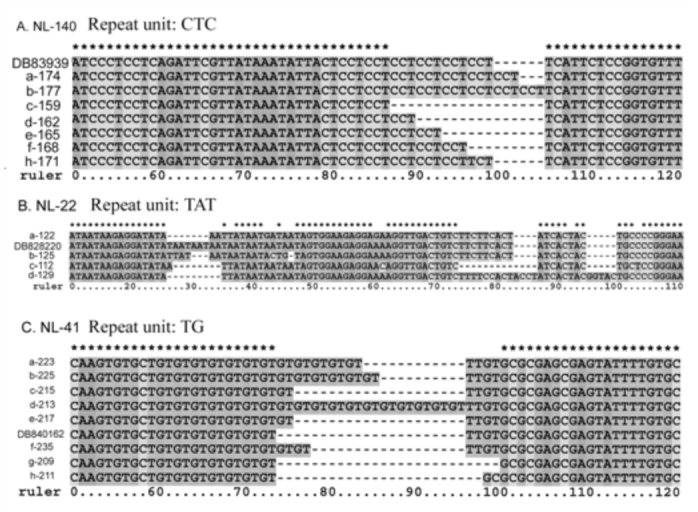
Part of Allele sequences for three microsatellites: A: NL-140, B: NL-22, C: NL-41. High quality figures are available online.

48 out of the total 78 alleles were sequenced successfully. The frequency of the 48 alleles accounted for 82.3% of the total alleles detected in populations genotyping. Rare alleles that existed in heterozygote form were difficult to sequence. By aligning the sequences in each locus, all the sequences we obtained were confirmed as the objective unigenes. Parts of aligned sequences are shown in Figure 3. Four types of microsatellite mutation patterns were found. First, the fragment length differences mainly came from the number variation of repeat motif. Second, imperfect repeats were observed in some alleles of the loci NL22, NL121, NL122, NL161 and NL177. Third, the two microsatellites coexisting in the same sequence contributed to length differences. At the loci of NL122, NL157, NL158, and NL167, there were more than one microsatellite which were not identified by software due to rare numbers (2–3) of repeat motifs in data mining. Finally, insertions/indels in the regions flanking the repeat motif also occurred on occasion at one allele of NL22 and NL162 separately. The frequencies of the two alleles were, however, very low (NL22: 0.025; NL162: 0.064).

## Discussion

### SSR frequency and distribution

The EST-SSR frequency was 1SSR/13.0 kb with nearly 5.6% of unigenes containing at least one SSR for the *N. lugens* transcriptome, which was similar to the ratios in wheat (1 SSR/15.6 kb) (Kantety et al. 2002) and barley (1SSR/6.3 kb) (Thiel et al. 2003). However, the EST-SSR frequency for the *N. lugens* transcriptome was smaller than in the pea aphid (1SSR/3.0 kb) and the green pea aphid (1SSR/3.6 kb) (Weng et al. 2007). The apparent frequency of EST-SSR is highly dependent on the search criteria applied and the type of SSR motif. Because di-, tri- and tetra-nucleotide motifs are most commonly targeted for marker development, we ignored mononucleotide motifs. In addition, we used higher search criteria than Weng et al. (2007). As a result, the EST-SSR frequency for the *N. lugens* transcriptome was smaller than the pea aphid and the green pea aphid.

Up to now, DNRs and TNRs were mostly reported in plants (Table 1), but the dominant repeat motifs were not in consistently observed in other organisms. In the *N. lugens*, based on our results, TNRs (65.2%) were the leading repeat motif, followed by DNRs (29.7%). This result was similar to the pea and green pea aphid repeat motifs from Weng et al. (2007), but contrasts with results from Meglécz et al. (2007) in which trinucleotide repeats were the third most abundant repeat motifs after mono- and dinucleotide repeats in all 10 insect species examined. Two reasons may contribute this difference. First, in Meglécz's study, genomic DNA sequences were used for SSR search, although, the EST sequences, probably located in the coding regions were used in both Weng et al. (2007) and our study. To avoid frameshift mutations in the coding regions, trinucleotide repeats motifs become the most abundant repeat motifs. Second, higher search criteria for the trinucleotide repeats motifs type SSR were used in our study (five repeats) than by Meglécz et al. (2007) (four repeats). In addition, AG/TC was the predominant DNR and the CG motif was absent. Other researchers have also found the AG/CT motif to be one of the most abundant DNRs (Kantety et al. 2002; Wang et al. 2007). That may be due to the fact that a di-nucleotide motif can represent multiple codons depending on the reading frame and can translate into different amino acids. Thus the GA/CT motif can represent GAG, AGA, UCU and CUC codons in an mRNA population and translate into the amino acids Arg, Glu, Ala and Leu respectively. Ala and Leu are present in proteins at high frequencies of 8% and 10%, respectively. AAG/CTT was the most common TNR present in the *N. lugens*, and AAAT/ATTT the most common TTNR. The frequencies of TNRs and TTNRs in different organisms vary widely. The SSR frequency may help select the most suitable probe in the future when we develop SSR from the genome using traditional methods.

### Characteristics of the EST-SSRs

High—resolution fingerprinting for population genetic studies requires a large number of moderately polymorphic microsatellites. Hence we tested the utility of our EST-SSRs, evaluating the polymorphism with 12 primers in a testing panel of wild *N. lugens.* Those samples were preserved in absolute ethanol and stored at -70^°^C for one year. DNA was extracted using a Gomi's protocol and stored for 2 months at -20°C. These DNA samples were used to select markers suitable for large-scale testing of an easy extraction method that avoids the costs and labor associated with more elaborate extraction methods. Unigenes containing SSRs from unknown gene groups identified by the blast search were used to design primers using Primer 5.0. Thus, SSRs in these sequences may avoid selective sweeps and possess a neutral character. In addition, there are two advantages when the annealing temperature of each pair of primers is set at 55°C. First, high annealing temperatures can suppress nonspecific amplifications, which lead to unclear banding patterns. Second, the same annealing temperature is well suited for fast multiplex-PCR, which can greatly reduce labor, time and cost.

Microsatellites sequencing revealed complex mechanisms of mutations of the 12 microsatellites loci. Supporting research has also reported interspecific and intraspecific size variation at microsatellite loci that was caused solely by number variation in the primary tandem repeat unit (Angers and Bernatchez 1997; Buteler et al. 1999; Colson and Goldstein 1999). For example, Matsuoka et al. (2002) reported complex mutation patterns of the maize (Zea *mays ssp. Mays*) and had minimal effects on analysis of interand intraspecific variation. Complex mutation of microsatellite may be a universal phenomenon in organisms. However, this research, shows insertions/indels in the regions flanking the repeat motif occuring in low frequencies. The fragment length differences were mainly a result of the variation of tandem repeat units that contained imperfect repeats and more than one type of repeat.

In spite of the complex mutation patterns, the 12 newly identified EST-SSR markers tested proved to range from moderately to highly polymorphic except the locus of NL157. In the ZJ and JS populations, two (NL22 and NL177) of the 12 microsatellites displayed a significant departure from Hardy-Weinberg equilibrium due to significant heterozygote deficiency. Two reasons may contribute to the heterozygote deficiency. First, null alleles may be the main reason of the heterozygote deficiency. Micro—Checker software confirmed the presence of null alleles at the two loci in the population that were a departure from Hardy-Weinberg equilibrium. Another important factor was inbreeding. The *N. lugens* samples collected for this study included some brachypterous (short-winged) individuals. Unlike the macropterous form, the brachypterous form has low migration ability, and may be more likely to inbreed. From the analysis of the three *N. lugens* populations, we found that the pairwise genetic differentiation between populations sampled in different years was higher than that between populations sampled in the same year. This may be the result of different origins of *N. lugens* in different years. However, given that only three populations were analyzed in this study, developing a clearer migration path of the *N. lugens* requires analysis of more populations and more powerful analytic methods.

**Table 1.**
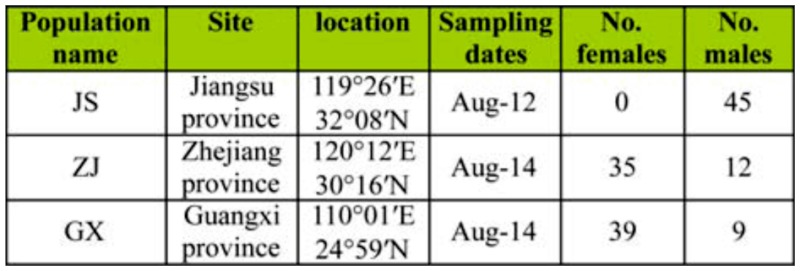
The *Nilaparvata lugens* populations used in this study.

**Table 2.**
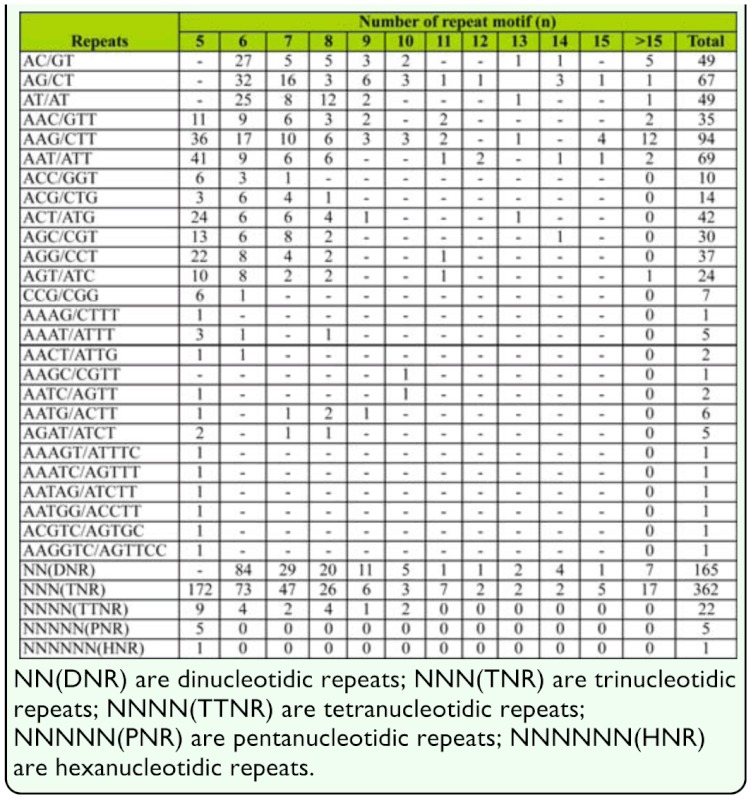
Frequency and distribution of SSRs in the analyzed the *Nilaparvata lugens* ESTs.

**Table 3.**
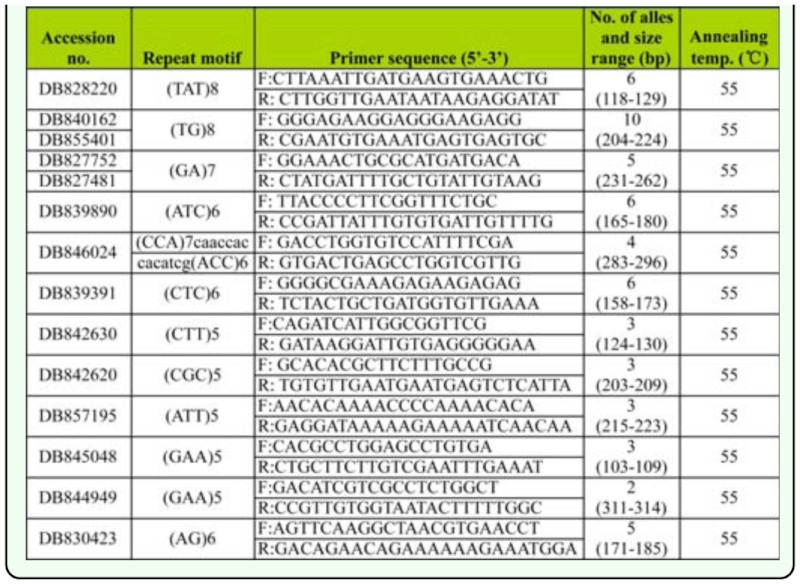
Primer sequence and character of 12 microsatellite locus isolated in the *Nilaparvata lugens.*

**Table 4.**
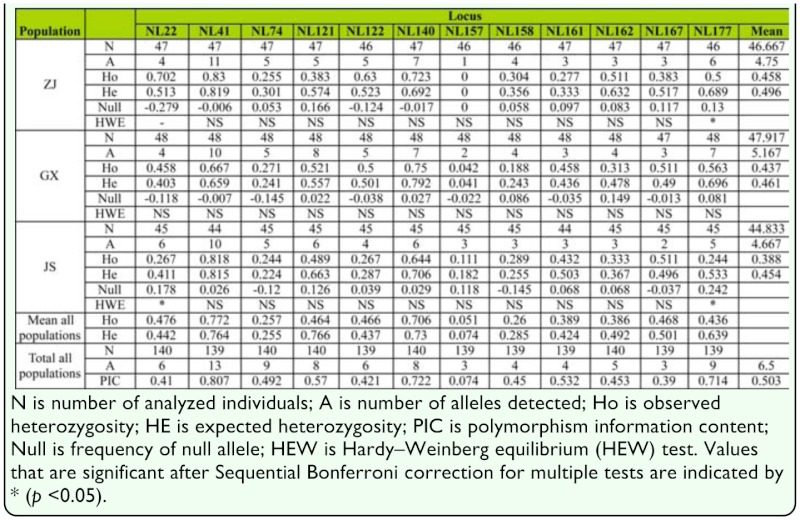
Population genetic parameters for each locus and population of the *Nilaparvata lugens.*

## Abbreviations

**EST**,: expressed sequence tag;
**GX**,: Guangxi province,
**JS**,: Jiangsu province;
**SSR**,: simple sequence repeats;
**ZJ**,: Zhejiang province
